# Back to the future: new target-validated Rab antibodies for evaluating LRRK2 signalling in cell biology and Parkinson's disease

**DOI:** 10.1042/BCJ20170870

**Published:** 2018-01-05

**Authors:** Patrick A. Eyers

**Affiliations:** Department of Biochemistry, Institute of Integrative Biology, University of Liverpool, Liverpool L69 7ZB, U.K.

**Keywords:** antibodies, LRRK2, monoclonal, Parkinson's disease, phosphorylation, Rab proteins

## Abstract

The addition of phosphate groups to substrates allows protein kinases to regulate a myriad of biological processes, and contextual analysis of protein-bound phosphate is important for understanding how kinases contribute to physiology and disease. Leucine-rich repeat kinase 2 (LRRK2) is a Ser/Thr kinase linked to familial and sporadic cases of Parkinson's disease (PD). Recent work established that multiple Rab GTPases are physiological substrates of LRRK2, with Rab10 in particular emerging as a human substrate whose site-specific phosphorylation mirrors hyperactive LRRK2 lesions associated with PD. However, current assays to quantify Rab10 phosphorylation are expensive, time-consuming and technically challenging. In back-to-back studies reported in the *Biochemical Journal*, Alessi and colleagues teamed up with clinical colleagues and collaborators at the Michael J. Fox Foundation (MJFF) for Parkinson's research to develop, and validate, a panel of exquisitely sensitive phospho-specific Rab antibodies. Of particular interest, the monoclonal antibody-designated MJFF-pRAB10 detects phosphorylated Rab 10 on Thr73 in a variety of cells, brain extracts, PD-derived samples and human neutrophils, the latter representing a previously unrecognised biological resource for LRRK2 signalling analysis. In the future, these antibodies could become universal resources in the fight to understand and quantify connections between LRRK2 and Rab proteins, including those associated with clinical PD.

## Introduction

Parkinson's disease (PD) affects ∼1 in 100 people over the age of 60, with the number of sufferers predicted to increase to >10 million in the world's most populated nations by 2030 [1]. In the U.K. alone, over 125 000 people are living with the disease, which is characterised by a range of symptoms, including neurodegeneration associated with the progressive loss of dopamine-containing neurones and a broad range of debilitating physical disorders [2]. Various cellular mechanisms have been proposed to contribute to the localised neuronal death that occurs in the substantia nigra of PD brains. These include protein folding/aggregation defects generating α-synuclein-rich Lewy bodies, abnormal protein phosphorylation/ubiquitination, aberrant intracellular trafficking and oxidative stress coupled to mitochondrial dysfunction [3]. None of these potential mechanisms are mutually exclusive, so piecing together the signalling pathways that might be manipulated pharmacologically for broad PD patient benefit remains an important goal. Moreover, the unpredictable, and often short-lived, benefits of classical pharmacological interventions that counteract effects of dopamine depletion in PD would benefit from stratified approaches in order to take advantage of druggable lesions in different patient groups [4].

In the genetic context, ∼20 genes have been directly linked to PD [5], although these currently account for only ∼5% of all familial cases [6]. An important contributing factor within this population is the human genomic PARK8 locus on chromosome 12 [7], which encodes the multi-domain polypeptide termed LRRK2 (leucine-rich repeat kinase 2). LRRK2 contains an array of structural signalling motifs alongside protein kinase and GTPase-containing domains [8,9]. Through a combination of poorly defined enzymatic mechanisms, LRRK2 regulates signal transduction and protein : protein interactions [10] cytoskeletal dynamics [11], Rab-dependent vesicular trafficking [12] and cellular ciliogenesis [13]. Interestingly, the majority of evidence put forward to explain how LRRK2 contributes to PD favours abnormal phosphorylation-dependent signalling properties of mutated enzymes [10]. This includes synergistic contributions between activating kinase domain mutations, such as the G2019S substitution in the highly conserved DFG motif found in eukaryotic protein kinases [14] and the tandem Roc/COR (Ras of complex proteins/C-terminal Of ROC) GTPase-containing domain (e.g. R1441G/C). The potential to treat groups of PD patients affected by this spectrum of LRKK2-associated mutations [5,15] has made the LRRK2 kinase domain an important new drug target [16].

## Rab GTPase proteins are endogenous LRRK2 substrates

To establish a clear route to the clinic, phosphorylated LRRK2 substrates have been sought as potential biomarkers to monitor ‘on-target’ LRRK2 drug engagement and clinical responses. However, until recently, a lack of validated phosphorylated substrates represented a general roadblock in the field. Works from several groups have established that CNS-active, target-validated small molecule LRRK2 kinase inhibitors have the potential to interfere with LRRK2-based signalling in PD [16,17]. This potentially includes LRRK2-mediated autophosphorylation on a variety of Ser residues [10,18–21], which have been suggested to represent biomarkers for LRRK2 activity (and might also target it for ubiquitin-mediated degradation) within the emerging LRRK2 signalling ‘module’ [22]. However, and as reiterated in the new studies from Alessi and colleagues [23–25], with the probable exception of pS935 [19] and pS1292 [18], these phosphorylated residues are potentially unreliable as robust biological readouts to report LRRK2 signalling in cell-based models and patient samples [26].

Recent mass spectrometry-based phosphoproteomics uncovered multiple Rab GTPases as direct physiological substrates of LRRK2 [12,13,19]. These findings are interesting for several reasons, not least due to the potential for phosphoregulation of the Rab proteins, which control multiple aspects of eukaryotic intracellular membrane trafficking [27]. Moreover, the protein kinase PINK1 (PTEN-induced putative kinase 1; PARK6) and the ubiquitin E3 ligase Parkin (PARK2) are also linked to PD signalling upstream of Rab GTPases [28], and the Rab (Ras-related proteins in brain) GTPase-related protein RAB7L1 (PARK16) functions in a vesicular trafficking pathway downstream of LRRK2 [29]. Consistently, the Rab proteins were also linked to PD through the analysis of α-synuclein (PARK1/4) and vesicular trafficking in both yeast and human models [30].

The important discovery — human LRRK2 directly phosphorylates a wide variety of Rab proteins, coupled with the recent finding that Rab29 is itself a Golgi LRRK2 activator [31] — opens up a series of new opportunities for researchers. For example, the evaluation of phosphosite preferences reveals that LRRK2-catalyzed phosphorylation occurs on a conserved Ser or Thr residue in 14 separate Rab proteins located within the nucleotide-sensing switch II region of the GTPase domain. These amino acids (Glu-Arg-Phe/Tyr-Arg/His-Ser/Thr-Hyd, where Hyd=hydrophobic, and Ser/Thr is the site of LRRK2 phosphorylation) conform to the broad LRRK2 substrate consensus motif previously defined with peptide substrates [32]. The identification of such a wide variety of Rab proteins as physiological LRRK2 substrates means that one obstacle for evaluating LRRK2 signalling output has essentially been overcome, with rapid, quantitative and reproducible Rab phosphorylation assays becoming a high priority on the research menu.

## Towards a comprehensive Rab antibody research toolbox

Now, in a major follow-up to these studies reported in back-to-back papers published in the *Biochemical Journal* [23,24], Alessi and co-workers have developed, validated and exploited exquisitely sensitive polyclonal and monoclonal phospho-specific Rab antibodies. These include a new monoclonal antibody for rapid detection of picogramme amounts of Rab10 phosphorylated by LRKK2 at Thr73 (MJFF-pRAB10). Remarkably, the unique His residue at the −1 position relative to the phosphorylation site in Rab10 appears to have aided creation of this highly specific reagent, which avoids cross-reactivity with very similar Rab proteins in this region, including Rab8A/B and Rab35 [23]. Importantly, Rab10 phosphorylation at Thr73 increases appropriately in a panel of mouse knockin cells containing hyperactive PD-associated LRRK2 proteins and is prevented by prior brief exposure to chemical LRRK2 inhibitors [23,24]. Since the detected site of Rab10 phosphorylation is conserved in mammalian orthologues, this reagent is ideal for analysing Rab10 phosphorylation under a variety of experimental conditions. For other Rab phospho-specific antibodies reported (e.g. MJFF-pRAB8), ‘pan-Rab’ phosphospecificity was clearly evident, and as confirmed by the authors, the ability of these antibodies to selectively immunoprecipitate a variety of phosphorylated Rab proteins, including Rab8A, Rab10 and Rab35, is also of considerable experimental utility [13,23]. As summarised below, these tools have significant potential to become work-horses in the signalling field, because they can be exploited to reveal activation or inhibition of LRRK2-catalysed phosphorylation in a wide variety of basic and clinical settings.

Initially, the authors focused on raising peptide-based antibodies in a variety of immunogenic hosts, with the common goal of recognising specific phosphorylated Rab proteins. Readers are referred to Tables 1 and 2 of the Lis paper [23] for details of the very broad range of antibodies analysed, and their potential utility in different biological scenarios. Depending on the antibody employed, the reagents can be used either as classical ‘primary antibodies’ in immunoblotting-based studies (specific protocols are described in the Materials and Methods) or for Rab protein immunoprecipitation from defined lysates. Another useful tool reagent discussed is a new Rab10 monoclonal antibody, termed MJFF-total Rab10^clone-1^, which is superior to all previously described Rab10 antibodies in terms of specificity and sensitivity [23]. Indeed, an overarching strength of the experiments reported in these papers is the rigorous analytical work undertaken, including biochemical, tissue-based and knockout/knockin approaches for antibody evaluation. These experiments have led to the creation of a benchmarked set of tools from which all researchers in the LRRK2/Rab field can benefit, since the considerable time invested has resulted in a diverse panel of sensitive and highly selective antibodies.

## New LRRK2 : Rab10 analytical opportunities in human neutrophils?

A biological role for LRRK2 in the immune system has previously been described [33], including marked increases in proinflammatory cytokines in an LRKK2 PD model [34]. However, previous reports did not evaluate LRRK2 mRNA or protein expression in circulating neutrophils. Interestingly, both LRRK2 and Rab10 protein were found to be abundant in purified neutrophils (and monocytes) at significantly higher levels than in the peripheral blood mononuclear cell population, which was examined side-by-side [24] and in an independent study [19]. Excitingly, the highly abundant neutrophil source was purified and employed for quantification of exogenous LRRK2 signalling before and after LRRK2 inhibitor exposure *ex vivo*, using Rab 10 phosphorylation as an LRRK2 biomarker in neutrophils isolated from a variety of human subjects [24]. It is logical to speculate that these antibodies might therefore find future utility in PD patient-derived neutrophils, potentially as predictive or responsive biomarkers for reporting LRRK2 activity in appropriately powered cohorts of subjects, an analysis the authors are careful to point out has not yet taken place [24]. As a priority, the Rab antibody panel should also be tested for immunofluorescence-based evaluation of Rab phosphoprotein levels in various fresh or archived cells, tissues and/or patient-derived samples.

Finally, in an experiment to delight biochemical aficionados, Fan et al. demonstrate that to isolate, and quantify, LRRK2 and Rab10 from the protease-rich neutrophil environment, an extract *must* be prepared in the presence of an irreversible serine protease inhibitor such as diisopropylfluorophosphate (DIFP). Under these conditions, endogenous Rab 10 Thr35 phosphorylation can also be readily detected in neutrophils by immunoblotting [24], with superior signals obtained when compared with those of the (more costly) Phos-tag-based polyacrylamide electrophoresis procedure [25]. Moreover, the exclusion of DIFP in isolation buffers leads to rapid LRRK2 proteolysis, yet its inclusion permits stable LRRK2 activity to be quantified even after 24 h of sample storage at room temperature. A further impact from this work could therefore be the creation of a formalised protocol for the collection and storage of neutrophils from appropriate patients for subsequent LRRK2/Rab10 analysis. The optimisation of a rapid ELISA-based assay to measure the levels of Rab10 phosphorylation in samples might be considered a future priority in this context.

## Conclusion

Several predicted impacts of these new studies are emphasised. The first is the creation of a unique, and very wide-ranging, consolidated set of pan and specific Rab antibodies that can be exploited, and perhaps refined, by pertinent researchers worldwide. They include polyclonal and monoclonal antibody reagents, the latter available for a prolonged period of time in large amounts, an important consideration for future translational appraisal. Of equal importance, many of these reagents are, or will soon be, available either commercially or collaboratively. This published panel [13,16,19,23,24,35] can therefore be employed alongside other tools to interrogate LRRK2 signalling and Rab protein phosphorylation via conventional immunological procedures. However, the utility of these reagents might extend well beyond this rather specific research area, and their general availability could influence current and future generations of research scientists seeking to uncover LRKK2 and Rab biology associated with health and disease. For example, they could be exploited as basic tools to probe physiological events associated with human Rab-based phosphorylation in the context of organelle-based trafficking, including under conditions where endogenous LRRK2 signalling contributes to Rab phosphorylation in the absence of LRRK2 mutation (i.e. as part of a normal regulatory cycle). It is notable that the LRRK2-phosphorylated motif is also very highly conserved in Rab proteins such as Rab10 from yeasts to worms, flies and vertebrates. In addition, Ras-related Sec4p, the product of the budding yeast SEC4 gene, whose discovery in 1987 established the Rab field [36], shares an identical switch II region with human Rab8A/B, meaning that phospho-specific antibodies designed to recognise these human proteins might also be useful to evaluate whether Sec4p phosphorylation occurs at this site in model organisms from yeast to flies, another useful LRKK2 model of PD [37]. Secondly, the fruits of these labours now permit the careful analysis of Rab signalling in a variety of systems specifically created to model and analyse PD, most notably dynamic Rab10 phosphorylation in the context of PD-associated LRRK2 mutations. It will also be illuminating to extend the analysis of Rab10 (and perhaps Rab8A/B, whose shared motif for LRRK2 phosphorylation is more challenging to probe specifically) phosphorylation in a much larger panel of control and PD brains, focusing on the substantia nigra and striatum, in addition to the cingulate cortex analysis reported here [23]. Thirdly, and as explained in some detail by Fan et al. [24], the feasibility, and inherent practicality of analysing LRRK2 and Rab10 phosphorylation in peripheral (non-CNS) cells, exemplified by the readily isolated neutrophil subpopulation, could lead to changes in PD patient sampling protocols. The ability to quantify pharmacodynamic changes in Rab protein phosphorylation before, during and after clinical intervention is an obvious outcome in this context.

These studies represent the latest outputs from a long-term collaboration between basic and clinical LRKK2 scientists, and the MJFF charity created and inspired by Michael J. Fox, whose memorable time-travelling experiences alongside inventor Christopher Lloyd in the ‘Back to the Future’ films influenced generations of (somewhat less ambitious) research scientists. Looking forward to the future, we can safely predict that this collection of validated antibodies, many of which bear his name, are likely to have important roles in basic and clinical settings, including the specific analysis of Rab phosphorylation in PD patients.

## Abbreviations

DIFP: diisopropylfluorophosphate
LRRK2: leucine-rich repeat kinase 2
MJFF: Michael J. Fox Foundation
PD: Parkinson's disease
RAB: Ras-related proteins in brain
